# Chlorhexidine, clotrimazole, metronidazole and combination therapy in the treatment of vaginal infections

**DOI:** 10.25122/jml-2019-0160

**Published:** 2021

**Authors:** Shahla Mirzaeei, Maryam Zangeneh, Firoozeh Veisi, Somayeh Parsa, Maryam Hematti

**Affiliations:** 1. Research Center, School of Pharmacy, Kermanshah University of Medical Sciences, Kermanshah, Iran; 2. Department of Obstetrics and Gynecology, School of Medicine, Kermanshah University of Medical Sciences, Kermanshah, Iran; 3. Research Committee of Students, Kermanshah University of Medical Sciences, Kermanshah, Iran; 4. Clinical Research Development Centre, Imam Reza Hospital, Kermanshah University of Medical Sciences, Kermanshah, Iran

**Keywords:** vulvovaginal candidiasis, bacterial vaginitis, nonspecific vaginitis, chlorhexidine vaginal gel

## Abstract

This was a clinical trial study that aimed to investigate the efficacy of vaginal chlorhexidine gel in the treatment of vulvovaginal candidiasis, bacterial vaginosis, and nonspecific vaginitis. The study population included patients who complained of vaginal discharge and presented to our University Gynecology Clinic. The data were analyzed using the Statistical Package for the Social Sciences (SPSS) software. The student t-test and Mann-Whitney U test were used to analyze the quantitative and ordinal data, respectively. In order to analyze the qualitative data, the Chi-square or Fischer’s exact tests were used. The mean satisfaction score in the vulvovaginal candidiasis patients who received chlorhexiine vaginal gel was 9.06 and 8.29 in the patients who received clotrimazole vaginal cream. The Mann-Whitney test did not show a statistically significant difference between mean scores of VAS in these two groups with vulvovaginal candidiasis (P=0.027). Among the patients with bacterial vaginosis, the mean satisfaction score was 8.91 in the chlorhexidine vaginal gel group and 8.72 in the metronidazole tablet group (P=0.607). In the nonspecific vaginitis group, the mean satisfaction score was 8.83 in the chlorhexidine vaginal gel group and 9.17 in the combination group (metronidazole + clotrimazole vaginal cream)(P=0.401). The highest mean visual analog scale score (VAS) score was documented in the combination therapy group. We found that chlorhexidine vaginal gel is a more effective method for the treatment and improvement of vaginal infections. The benefits of chlorhexidine gel have a positive therapeutic effect as a single drug in nonspecific vaginitis, rather than simultaneous administration of two agents.

## Introduction

Vaginal bacterial infections have gained attention due to the increased prevalence of urinary tract infections (UTIs) and sexually transmitted diseases (STDs) [1]. Bacterial infections are among the most common causes of vaginal discharge during the reproductive age [2]. Vaginitis is also the most common cause for women to visit gynecology clinics [3].

Vulvovaginal candidiasis (VVC) is the most common vaginitis in women. It is estimated that about 75% of women develop VVC at least once in their lifetime. This vaginitis involves increased colonization and exacerbation of Candida (85% to 90%, Candida albicans) invasion to the epithelial cells of the lower genital tract [4]. Bacterial vaginosis (BV) refers to the relative lack of vaginal endogenous lactobacilli and synergistic anaerobic microbial growth in the vaginal epithelium. Although vaginal bacterial infections are often asymptomatic, they are one of the common causes of vaginitis and the main cause of women's need for medical care [5]. There is also a type of vaginitis that does not meet the criteria presented above but involves a combination of some of the features of VVC and BV. This group is called nonspecific vaginitis.

The usual treatment recommended by the Center for Disease Control and Prevention (CDC) for VVC includes vaginal clotrimazole (the most commonly used treatment) or oral antifungal drugs such as fluconazole prescribed as a single 150 mg dose. The common treatment for BV is oral or vaginal antibiotics such as metronidazole or clindamycin [6, 7]. The treatment used for nonspecific vaginitis includes oral metronidazole plus vaginal clotrimazole. Although these treatments have been effective in the short term, they are not effective in preventing infection recurrence in the long term in at least half of patients [8].

Several alternative treatments, including antiseptics, have been proposed. Antiseptics have been used to treat vaginal infections in the last century. Similar to antibiotics, antiseptics cause the destruction of vaginal anaerobic bacteria due to vaginal infections, which results in the regeneration of endogenous lactobacilli. Antiseptics usually have a broad spectrum of effects. They act through the mechanism of destruction of the cell wall, eradicating the bacteria. Accordingly, there are very limited reports of resistance to these factors [9].

Aerobic infections are another common condition caused by abnormal vaginal flora, which is one of the most important factors in pregnancy complications, such as an increase in amniotic fluid infections, premature rupture of membranes, and premature labor [10]. In combination infections, single-drug treatment is ineffective, while treatment with broad-spectrum antibacterial and antifungal agents, such as chlorhexidine digluconate, may be very promising for rapid treatment [11].

Several drug delivery systems are used to treat vaginal infections [12]. Old vaginal formulations (suspensions, pills, creams, and solutions) remain in place for a short period due to the washing of the vaginal fluid by physiological secretions. Vaginal drug delivery systems that adhere to mucosae such as pills and gels stick to the vaginal mucus and prevent the drug from washing. Pills and some vaginal delivery systems, such as creams and suspensions, are accompanied by irregularities and formulation leakage that have caused discomfort and disapproval of consumers [13, 14].

Current treatments for such infections include antifungal and antimicrobial drugs, which are narrower for their specificity. Of course, its specificity is due to the fact that these drugs are systemically defined, so the pharmacological side effects should be as few as possible, which is not important for topical drugs. The side effects of a topical drug are limited to local toxicity. Regarding chlorhexidine, this medicine is used orally, and there is no local toxicity that prevents it from being consumed. For this reason, it has more potential in terms of performance, price and side effects than similar preparations. In addition, since chlorhexidine is classified as a topical antiseptic, it is categorized as an over-the-counter (OTC) drug and is easily available to patients. Also, studies have shown that chlorhexidine digluconate is very effective in reducing the E. coli and Candida albicans microbial load [15].

Since vaginal chlorhexidine gel has not been studied in Iran and the wide spectrum of organisms that chlorhexidine can be used for, it is a necessity to study this agent in vaginal infections. Therefore, this study aimed to compare the efficacy of vaginal chlorhexidine gel vs. routine treatments in the treatment of VVC, BV, and nonspecific vaginitis.

## Material and Methods

In this clinical trial, the study population consisted of female patients who presented to our University Gynecology Clinic complaining of vaginal discharge. We used the convenience sampling method. The estimated sample size was calculated considering the 95% confidence level and power of 80% and assuming a recovery percentage of metronidazole and vaginal clotrimazole as 93% and 74%, respectively [11]. The minimum required sample size was calculated as 34 patients in each group. In order to increase the accuracy of the study, we included 34 patients in the VVC group, 41 patients in the BV group, and 36 patients in the nonspecific vaginitis group (a total of 111 patients).

In order to determine VVC (group 1), clinical examinations of patients who complained of pruritus, burning, or vaginal discharge was done. The vaginal pH was measured by the tape method. The criteria to determine VVC were as follows: 1) vaginal discharge (thin to thick secretions), vaginal ulcers, dyspareunia, vulvar burning sensation, dysuria, and observation of erythema and edema of the vulva, erythematous vagina with white, thick and sticky secretions, as well as pustular or papular lesions; 2) vaginal pH, which is usually normal (less than 4.5) in VVC patients; 3) fungal components (budding yeasts and mycelium) are seen in 80% of patients; 4) negative Whiff test; 5) even in the absence of fungal components that can be detected by microscopy and in the case of normal pH, the results of the study of the prepared slide with normal saline and increased erythema in the vulvovaginal area, a possible diagnosis of VVC was suggested, and the patients were included.

In order to determine patients with BV (group 2), gynecologic examinations were conducted for women who presented with vaginal discharge, vaginal burning sensation, or abnormal vaginal odor. The vaginal pH was measured using the tape method. A smear was prepared using a cotton swab from vaginal secretions; a drop of KOH was added, and a whiff test was performed. Another smear was prepared and examined by an Obstetrics and Gynecology resident to find clue cells. After examination and confirmation of vaginitis, which included at least three items of the Amsel criteria, patients were enrolled in the study. The Amsel criteria include the followings:

1. A whitish-gray homogeneous discharge that covers the vaginal wall;
2. Vaginal pH>4.5;
3. Positive Whiff-amine test (fishy odor when adding KOH to vaginal secretions);
4. Clue cells that constitute at least 20% of the epithelial cells in the wet saline sample.

In the third group, after the gynecological examination and failure to identify the characteristics of the first and second groups, the patient was defined as having nonspecific vaginitis.

### Interventional medications

Firstly, hydroxyethylcellulose, disodium hydrogen phosphate, sodium chloride, propylene glycol, and glycerol were bought from the Merck pharmaceutical company. The active ingredient of chlorhexidine was provided by the Naju pharmaceutical company and ethanol was supplied by the Merck Company. The culture media (soybean casein digest agar and soybean digest broth) was bought from Merck Company. In the next step, the pre-formulation process of the chlorhexidine drug was prepared at the pharmacy school of our university. These steps included determination of the physicochemical properties of the chlorhexidine antimicrobial agent, studying various polymers as the drug base, studying drug compatibility with the base polymer, determining the appropriate buffer for adjusting the pH of the product, determining the formulation, and appropriate package components. To control the pharmaceutical product, two types of evaluations were performed on the product:

1. Physicochemical control of the product (viscosity test, homogeneity of the content, evaluation of the active ingredient);
2. Microbiological control (evaluation of the product on two types of microorganisms, including Staphylococcus aureus and Pseudomonas aeruginosa and Candida albicans).

After the preparation of this drug, tests were carried out to evaluate its physicochemical and microbial qualities. A sustainability test was also carried out, and it showed that the drug was completely stable in the long term (one year). After microbial and physicochemical tests, confirmation of chlorhexidine gel, and performing a pilot study, it was used in this study.

In the first group (VVC, 34 patients), clotrimazole vaginal cream (a1) or chlorhexidine vaginal gel (b1) were administered alternatively. In the second group (BV, 34 patients), chlorhexidine vaginal gel (a2) or metronidazole tablets (b2) were administered alternatively. In the third group (nonspecific vaginitis, 34 patients), chlorhexidine vaginal gel (a3) or combination therapy (clotrimazole vaginal cream and metronidazole tablets (b3) were administered.

Vaginal preparations were used using a vaginal applicator at bedtime for 5 days. Two doses (500 mg in total, 250 mg each tablet) of metronidazole tablets were used every 12 hours for 5 days.

As this was a double-blinded study, the drugs were administered randomly. The examiner and the personnel who delivered the drugs were unaware of the contents of the packages. After completing the 5-day course of the treatments, the patients were asked during the follow-up visits about the itching rate, burning sensations, side effects, and satisfaction with the treatments.

### Statistical analyses

The gathered data were analyzed using the Statistical Package for the Social Sciences (SPSS) software (v. 19.0). To analyze quantitative data, Leven’s test, as well as the independent-samples t-test, was used. We also used the Mann-Whitney U test o compare the ordinal data. For qualitative data, the Chi-square or Fischer’s exact test was used. The significance level was set at 0.05. A layered analysis was used for separate analysis of vaginitis groups.

## Results

Mean (SD) ages of the patients were 33.24 (±7.43), 33.41 (±6.87), and 31.86 (±6.83) years in the VVC, BV and nonspecific vaginitis groups, respectively. The results of the diagnostic criteria for VVC in the studied patients showed that there was no significant difference regarding cheese-like secretions, vulvar burning sensations, negative Whiff test, and observation of fungi under microscopy (P>0.05) between patients of the two groups of vaginal chlorhexidine and clotrimazole vaginal cream. Also, findings regarding the diagnostic criteria for BV showed no significant difference regarding the Whiff test (P=0.30), Nitrazine test (pH>4.5) (P=0.573), malodorous discharge (P=0.618) and clue cells (P=1.000) between the chlorhexidine vaginal gel and oral metronidazole groups.

As shown in Table 1, the most common side effect in the chlorhexidine vaginal gel and clotrimazole vaginal gel groups was vaginal burning and 14 patients (82.35%) experienced this side effect in each group. No nausea and vomiting were reported. Only one patient from the clotrimazole vaginal cream developed cutaneous lesions. Most patients experienced improvement of symptoms. Only two patients in the chlorhexidine vaginal gel and one patient in the clotrimazole vaginal cream groups did not report symptom improvement. There was no significant difference regarding vaginal burning between chlorhexidine vaginal gel and clotrimazole vaginal cream.

**Table 1.** The frequency distribution of side effects and improvement of symptoms in the vulvovaginal candidiasis, bacterial vaginosis, and nonspecific vaginitis groups.

Groups	Side effects	Chlorhexidine vaginal gel	Clotrimazole vaginal gel	Oral metronidazole	Combination therapy (oral metronidazole and clotrimazole vaginal gel)
**Vulvovaginal candidiasis (N=34)**	Vaginal burning	14 (82.35%)	14 (82.35%)	-	-
	Nausea	0	0	-	-
	Vomiting	0	0	-	-
	Cutaneous lesions	0	1 (5.88%)	-	-
	Improvement	15 (88.23%)	16 (94.11%)	-	-
**Bacterial vaginosis (N=41)**	Vaginal burning	10 (43.47%)	-	2 (11.11%)	-
	Nausea	0	-	1 (5.55%)	-
	Vomiting	0	-	1 (5.55%)	-
	Cutaneous lesions	0	-	0	-
	Improvement	23 (100%)	-	18 (100%)	-
**Nonspecific vaginitis (N=36)**	Vaginal burning	5 (27.77%)	-	-	6 (33.33%)
	Nausea	0	-	-	0
	Vomiting	0	-	-	0
	Cutaneous lesions	3 (16.66%)	-	-	0
	Improvement	18 (100%)	-	-	18 (100%)

In the BV group (41 patients), vaginal burning was higher in the chlorhexidine vaginal gel group (10 patients, 43.47%) compared to oral metronidazole (2 patients, 11.11%); P<0.05. Nausea and vomiting were reported in the oral metronidazole group. In both groups, no cutaneous lesions were reported, and improvement was seen in all patients (Table 1).

Of the 36 patients included in the nonspecific vaginitis group, 5 patients (27.77%) who received chlorhexidine vaginal gel and 6 patients (33.33%) who received combination therapy (oral metronidazole plus clotrimazole vaginal cream) reported vaginal burning. There were no cases of nausea and vomiting. Three patients from the chlorhexidine vaginal gel group developed cutaneous lesions (Table 1).

According to Table 2, the mean satisfaction score (analyzed using the visual analog scale – VAS) in patients from the VVC group was 9.06 and 8.29 for chlorhexidine vaginal gel and clotrimazole vaginal gel, respectively. There was no significant difference regarding VAS scores between these two groups (P=0.027). In the BV group, the mean satisfaction score was 8.91 in the chlorhexidine vaginal gel group and 8.27 in the oral metronidazole group (P=0.607). In the nonspecific vaginitis group, the mean satisfaction score was 8.83 for chlorhexidine vaginal gel and 9.17 for combination therapy (oral metronidazole and clotrimazole vaginal gel) (P=0.401). The most significant mean VAS score was seen in the combination therapy group (Table 2).

**Table 2.** Mean satisfaction scores based on the visual analog scale.

Groups	Clorhexidine vaginal gel		Vaginal clotrimazole			Oral metronidazole			Combination therapy (oral metronidazole and vaginal clotrimazole cream)		
	Mean	SD	Mean	SD	P-value	Mean	SD	P-value	Mean	SD	P-value
**Vulvovaginal candidiasis**	9.06	1.19	8.29	2.08	>0.05	-	-	-	-	-	-
**Bacterial vaginosis**	8.91	1.20	-	-	-	8.72	1.12	>0.05	-	-	-
**Nonspecific vaginitis**	8.83	1.29	-	-	-	-	-	-	9.17	1.04	>0.05

## Discussion

This study showed that the greatest recovery and satisfaction rates were seen in patients who received chlorhexidine vaginal gel. Besides vaginal burning, no other side effect was seen compared to other treatments, including clotrimazole vaginal gel, oral metronidazole, and combination therapy (oral metronidazole and clotrimazole vaginal gel).

According to the results of our study, there was no significant difference in the mean age between the three groups. The results of previous studies [16, 17] also showed that there was no significant difference between the mean ages of individuals, which is in agreement with the results of this study. Also, the results of the diagnostic criteria of VVC showed that there was no significant difference between patients in terms of the presence of cheese-like discharge, vulvar burning, negative Whiff test results, and fungal elements under microscopy between vaginal chlorhexidine gel and clotrimazole cream groups. In the BV group, there was no statistically significant difference between the diagnostic criteria of BV in terms of the results of the Whiff test, the Nitrazine test, and the presence of malodorous secretions between chlorhexidine vaginal gel and oral metronidazole groups. The results obtained in our study are similar to previous studies [18, 19] in identifying the type of BV. The results of other studies have shown that women who complain of a change in vaginal discharge can describe the type of discharge. In other words, there is a correlation between the symptoms of patients complaining of discharge and their clinical signs [20].

Based on the results obtained, in the VVC group, those who received chlorhexidine vaginal gel did not show any complications such as nausea and vomiting. Only one case of cutaneous lesions was observed. The burning sensation was reported in 3 patients. Improvement was observed in the majority of patients, and the satisfaction score of patients in this group was higher than that of clotrimazole vaginal cream recipients. In a previous study, 97 patients out of 99 patients with candidal vaginitis responded to the treatment with clotrimazole vaginal tablets [21]. In another study, triazoles such as fluconazole and terconazole as well as imidazoles such as clotrimazole, miconazole, itraconazole, ketoconazole and agents such as nystatin have been recommended for the treatment of candidal vaginitis [22]. In a study by Banaeian *et al.*, the effect of clotrimazole and honey gel was studied in two groups of women with vaginitis [23]. The authors reported that vaginal inflammation and secretion showed significant improvement in the clotrimazole group, and no complications were seen in either group. In the above-mentioned studies, improvement of symptoms has been reported with clotrimazole treatment. However, in our study, the effect of chlorhexidine vaginal gel was better than that of clotrimazole, which could be significant in treating vaginal infections.

The results of this study showed that in the BV group, the burning sensation was significantly higher in chlorhexidine vaginal gel in comparison to patients who received oral metronidazole treatment. Improvement of symptoms was seen in all patients. Also, one patient from the metronidazole group reported nausea and vomiting.

Jafarzadeh *et al.* stated that metronidazole was effective in the treatment of bacterial vaginosis. Despite the clinical improvements observed in women, metronidazole causes a change in the pattern of microbial flora. However, gram-negative bacterial anaerobes are still present after treatment [24], which is consistent with the results of our study. In this regard, the treatment of bacterial vaginosis may increase the risk of developing VVC, which should be considered.

Based on our results, improvement of symptoms in the nonspecific vaginitis group was reported in all patients, and no adverse effects such as nausea and vomiting were observed. Only 5 patients who received chlorhexidine vaginal gel and 6 patients who received the combination therapy group (clotrimazole vaginal cream + oral metronidazole) had a burning sensation that was not statistically significant. In various studies, metronidazole and clotrimazole treatments have been studied separately [17, 21]. Therefore, one of the strengths of our study is that we simultaneously examined the four drugs in vaginitis patients.

In a study by Mousavi *et al.*, 60 women aged 16 years and older whose vaginosis was characterized by standard Amsel and Gram staining were randomly assigned to receive oral clindamycin (300 mg twice daily for 7 days) or oral metronidazole (500 mg twice daily for 7 days). The authors showed that a significant number of patients who received metronidazole had a proven VVC diagnosed using the 10% KOH test [25], which is similar to the results of the present study.

In this study, we showed that treatment with chlorhexidine vaginal gel had good results in terms of the effect on bacteria and fungi in the preliminary stages. Nausea and vomiting were not observed in the patients, and only 3 patients (2.7%) had cutaneous lesions. The only significant side effect was a vaginal burning sensation (21.6%); however, this difference was not significant in other groups. These results are consistent with those obtained by Ohlsson *et al.* regarding burning in patients receiving chlorhexidine [26]. In our study, about 98.2% of patients who received vaginal chlorhexidine gel experienced improvement of symptoms. These results are consistent with the study by Molteni *et al.*, which showed a 93% improvement in using 0.5% vaginal chlorhexidine gel [11].

In the current study, the overall results showed that chlorhexidine vaginal gel was effective as a therapeutic treatment for improving and treating vaginal infections, including bacterial and fungal infections. Another advantage of chlorhexidine vaginal gel was its therapeutic effect as a single agent therapy compared to combination therapy in nonspecific vaginitis, making it easier for patients to receive treatment. For this reason, in terms of performance, price and side effects, it is a better option compared to the other treatments. In addition, since chlorhexidine is a topical antiseptic, it is categorized as an OTC drug; therefore, it is readily available to the patients, while the others are not OTC treatments. In addition, it is important to note that resistance to this antiseptic agent is much lower compared to antibiotics.

Considering the positive results of this study, we suggest that chlorhexidine vaginal gel should be used by other researchers with the hope of introducing this agent as a routine therapy option. Conducting more comprehensive studies can be helpful in increasing the accuracy of the results.

## Conclusion

Clinical effects of chlorhexidine vaginal gel, clotrimazole, oral metronidazole and combination therapy in the treatment of vaginal infections showed that chlorhexidine vaginal gel was the most suitable option in the treatment of vulvovaginal candidiasis and bacterial vaginosis and nonspecific vaginitis as a single agent compared to combination therapy.

## Acknowledgments

### Ethical approval

The approval for this study was obtained from the Ethics Committee of the Kermanshah University of Medical Sciences (approval ID: IR.KUMS.REC.1395.218).

### Consent to participate

Informed consent was obtained from the participants.

### Funding

Financial support of this work was provided by the Research Council of Kermanshah University of Medical Sciences (grant number: 96358).

### Conflict of interest

The authors declare that there is no conflict of interest.
